# Position-sensitive non-destructive detection of charged-particle bunches in low-energy beamlines

**DOI:** 10.1038/s41598-023-45798-6

**Published:** 2023-12-19

**Authors:** Stefan Ringleb, Markus Kiffer, Jonas K. C. Ballentin, Thomas Stöhlker, Manuel Vogel

**Affiliations:** 1 Friedrich Schiller-Universität Jena, 07743 Jena, Germany; 2 University of Vienna, 1090 Vienna, Austria; 3 Helmholtz-Institut Jena, 07743 Jena, Germany; 4 GSI Helmholtzzentrum für Schwerionenforschung, 64291 Darmstadt, Germany

**Keywords:** Experimental particle physics, Electronic and spintronic devices

## Abstract

We have developed and operated an electronic detection system for the non-destructive single-pass detection of bunches of charged particles in a beamline that allows for a measurement of their lateral position with respect to the central beamline axis on a shot-to-shot basis. It provides all features of our related development reported in Kiffer et al. (Rev Sci Instrum 90:113301, 2019), namely single-pass measurement of bunch length, kinetic energy and absolute charge, and is additionally designed to provide the lateral position of bunches with sub-mm accuracy. We show the setup, associated methods and provide characterizing measurements with bunches of highly charged ions in the keV regime of kinetic energy that demonstrate the capabilities and show a typical application.

## Introduction

Transport of charged particles from pulsed sources^1–3^ to a specific experimental location via a vacuum beamline is a common element of numerous setups and facilities^4–14^. Depending on the details of the experiment, different properties of the particle bunches during transport are sought to be monitored, most often this refers to particle kinetic energy, the amount of charge (number of particles) per bunch, its axial extension, lateral position (offset) relative to the central beamline axis and emittance^15^. These quantities can be measured destructively by a suite of methods and devices, however in many cases it is preferable or required to perform such measurements in a non-destructive way. This is true for example for rare species or when monitoring on a shot-to-shot basis is required. Such single-pass non-destructive detection and measurement is possible with regard to particle kinetic energy^16–18^ and the total amount of charge per bunch^17–19^. Similar methods are also in use for dedicated measurements of particle mass^20^. Recently, a system and method has been devised that in addition to particle kinetic energy and particle number allows for a determination of axial bunch extension from a single-pass non-destructive measurement^21^.

Presently, a system is introduced which furthermore allows for an accurate non-destructive determination of the lateral position of bunches in a beamline. It uses radial segmentation of the electrodes used for pick-up of image charges^22–24^ induced by the particle motion into four equal parts with individual signal processing. From a comparison of the respective induced signals regarding their intensity and timing, additionally the offset of the particle bunch from the central beamline axis can be determined in both lateral directions independently. This information allows for the use of ion optics^25,26^ to align the particle bunches to a specific lateral position, in particular to the central beamline axis. This is of considerable importance for example for particle injection into setups such as Penning traps, since any initial offset from the central trap axis leads to an undesired radial motion that is a potential source of particle loss from the trap^27^.

In the following, we describe the concept, mechanical setup, signal processing and methods for the determination of the desired bunch properties from the measured signals, and show its application to bunches of highly charged ions from an EBIT ion source^2^ injected into the Penning trap of the HILITE setup^6^ for future high-intensity laser-ion interaction studies.

## Concept and theory

Non-destructive sensing of charged particles relies on the well-known Shockley–Ramo theorem^22,23^ which relates the moving charges to image currents thus induced in nearby conducting electrodes that can be detected as a corresponding voltage signal. Note that the present concept of image-charge pick-up does not rely on electronic resonance with periodic signals like in the cases of particle oscillation in a trap^28^ or Schottky pickup^29^, but is a single-pass detection and allows for an analysis on a shot-by-shot basis.

**Figure 1:**
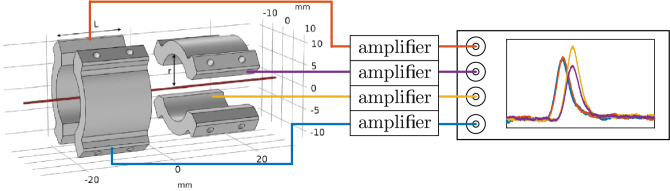
Concept of the position-sensitive charge counter. Two pairs of electrodes rotated by 90$^\circ$ with respect to each other. The centre of curvature of all electrodes is the central detector axis (red line). The electrode dimensions are $L={15}\textrm{mm}$ and $r={7.5}\textrm{mm}$. Each of the four signals is amplified by a dedicated amplifier and acquired with an oscilloscope.

**Figure 2:**
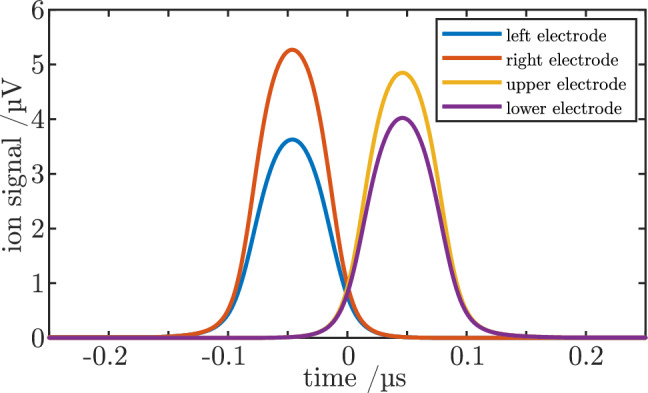
Calculated single-ion signals *U*(*t*) (voltages as a function of time) for the four pick-up electrodes of the detector. The differences in signal height between left and right and upper and lower electrodes measure the ion offsets in both lateral directions. The time difference between these two signal pairs measures the particle velocity.

A particle with charge *q* induces an image charge $q_{ind}$ that is given by

1 $$\begin{aligned} q_{ind} = -\Gamma (\vec {r}) \cdot q, \end{aligned}$$

where $\Gamma$ is the so-called *geometry function* that for a given electrode geometry depends only on the position $\vec {r}=(x_0,y_0,z_0)$ of the particle, as will be discussed below. Here, (*x*, *y*) is the plane perpendicular to *z* such that $(x_0=0,y_0=0)$ defines the central *z*-axis through the device (dark red line in Fig. 1). In each detector electrode, the induced charge $q_{ind}$ leads to a voltage signal as a function of time that is then amplified and recorded. For a particle passing the detector electrode along the *z*-axis, the expected signal as a function of time is a voltage peak as shown in Fig. 2, with the voltage starting at zero when the particle is (infinitely) far away, reaching a maximum at the axial centre of the detection electrode pair and dropping to zero again. The knowledge of the geometry function can be used to measure the bunch length, as has been detailed in^21^.

Generally, from such signals, information on the particle kinetic energy, the length of a particle bunch and the contained charge can be derived^17–19,21^. If the individual particle charge is known, this allows for a counting of particles with close to single-particle sensitivity^18,21^. However, implementations of such a detector that use radially symmetric electrodes (e.g. hollow cylinders) that are pervaded by the particle bunches can be shown to be insensitive to their lateral position^30^, thus being unsuited for a position measurement.

This can be alleviated by a segmentation of the pickup electrode such that the signals induced in its individual parts become dependent on the lateral particle position. Presently, this is done by an arrangement of two subsequent pairs of opposing half-cylinders with the second pair rotated by 90$^\circ$ with respect to the first pair as shown in Fig. 1.

This leads to four individual signals being induced by a single particle or bunch, the first pair of which resolves the *x*-direction (horizontal), while the second pair resolves the *y*-direction (vertical). The time delay between the two pairs of signals yields the particle velocity along *z* and hence the particle kinetic energy given that the particle species is known.

### Expected detector signals

The dependence of the expected single-particle signal on the particle position is quantified by use of the geometry functions $\Gamma$ of the electrodes used for signal pick-up. The function $\Gamma$ for any individual detector electrode is obtained from setting its potential to 1 V, with all other conducting surfaces set to ground, and using the FEM-solver ComSol multiphysics AC/DC package to find the resulting electrostatic potential $\Phi (\vec {r})$ for all relevant positions $\vec {r}=(x_0,y_0,z_0)$. The dimensionless value $\Gamma (\vec {r})$ is then defined as $\Phi (\vec {r})/(1\text{ V})$. This definition is identical to the one used previously in^21^.

Using the geometry function $\Gamma$ of any of the four detector electrodes, the expected signal voltage *U*(*t*) in the corresponding detection channel is given by

2 $$\begin{aligned} U(t) = -q_{ind}\cdot S = \Gamma \left( x_0(t),y_0(t),z_0(t) \right) \cdot q \cdot S, \end{aligned}$$

where *q* is the charge of the single particle and *S* is the sensitivity of the detection channel which is a characteristic value for each channel that has to be determined independently as will be discussed below. The minus sign inverts the measured signal such that a positive signal corresponds to a positive initial charge *q*. Since the magnitude of the signal does not depend on particle velocity, the present method can be used across a wide range of particle kinetic energies, limited only by the choice of detection electronics.

As an example, Fig. 2 shows the four time-dependent voltage signals induced by a single Ar$^{7+}$ ion with a kinetic energy of 2000 eV/q. The assumed sensitivity *S* is 1500 nV/e. To illustrate the effect of a lateral displacement from the central axis, the ion is offset from the axis by 1 mm in the horizontal and 0.5 mm in the vertical direction.

This leads to signal differences between the left and right electrodes and between the upper and lower electrodes that have magnitudes corresponding to the chosen offsets. The time difference between the left/right signal pair and the upper/lower signal pair is proportional to the ion velocity and can thus be used for a determination of ion kinetic energy.

### Lateral position determination

One can infer the particle position in any of the lateral directions *x* and *y* from a comparison of the signals in corresponding pairs of opposing detector channels. This is true as for each electrode the induced signal depends on the lateral particle distance to the detector axis by virtue of Eq. (1).

For a quantification of the lateral particle position, we use the area

3 $$\begin{aligned} A = \int _{-\infty }^{\infty } U(t) \text{ d }t \end{aligned}$$

of the signal in each detector channel as calculated from Eq. (2), i.e. we obtain values $A_{right}$, $A_{left}$, $A_{bottom}$ and $A_{top}$ for each lateral position $(x_0,y_0)$. From these, we calculate the dimensionless ’contrast ratios’ $\Omega _x$ and $\Omega _y$ which we have introduced to be independent of the ion number. It is defined by

4 $$\begin{aligned} \Omega _x = \frac{A_{right}-A_{left}}{A_{right}+A_{left}} \;\;, \;\;\; \Omega _y = \frac{A_{top}-A_{bottom}}{A_{top}+A_{bottom}}, \end{aligned}$$

again for each lateral position $(x_0,y_0)$. This results in two maps $\Omega _x(x_0,y_0)$ and $\Omega _y(x_0,y_0)$ of contrast values, one for the horizontal detector part and one for the vertical. Note that while the signal area *A* is proportional to the particle charge *q*, the contrast $\Omega$ is by definition independent of it.

**Figure 3:**
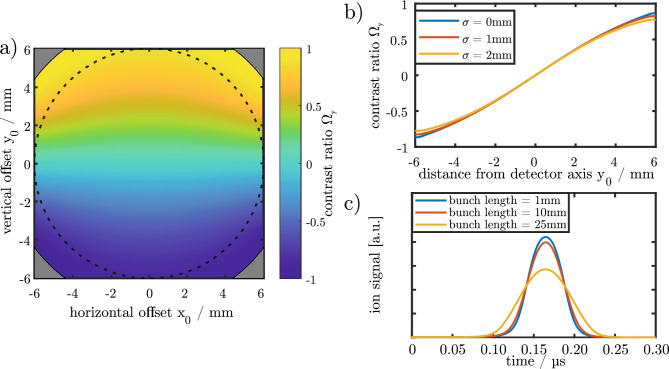
(**a**) Calculated dependence of the contrast ratio of the vertical electrode pair on the vertical and horizontal particle offsets from the central axis. The contrast ratio was calculated assuming no lateral extent of the ion beam. The dotted circle is the aperture of the detector with a diameter of 12mm and the gray corners are the electrodes. As desired, the vertical contrast ratio depends nearly linearly on the vertical particle position while it is almost independent of the horizontal particle position. (**b**) Influence of the finite bunch size. The contrast ratio decreases slightly with for lateral extended ion bunches. This effect is negligible in the detector centre. (**c**) Influence of the bunch length. Long bunches have a lower peak voltage while the area under the signal is independent of the bunch length.

As an example, Fig. 3a shows the map of the position-sensitive contrast value $\Omega _y(x_0,y_0)$ of the detector’s vertical part.

Close to the detector centre the vertical contrast value depends linearly on the distance in the *y*-direction and has almost no dependence on the *x*-direction, as desired. For the horizontal part of the detector, the same statement is true with *x* and *y* inverted. The contrast is zero for vanishing particle offset ($x_0=y_0$=0, center of the figure) and $\pm 1$ for maximum vertical and horizontal offset (corners of the figure).

From a measured pair of contrast values $(\Omega ^{(m)}_x,\Omega ^{(m)}_y)$, the actual offset ($x_0,y_0$) of a particle from the central axis can then be determined by use of the two calculated contrast maps $\Omega _x(x_0,y_0)$ (horizontal) and $\Omega _y(x_0,y_0)$ (vertical, as shown in Fig. 3a) and a simple algorithm that minimizes the quantity

5 $$\begin{aligned} (\Omega ^{(m)}_{x}-\Omega _{x}(x_0,y_0))^2 + (\Omega ^{(m)}_{y}-\Omega _{y}(x_0,y_0) )^2 \end{aligned}$$

which is minimum for the actual values of the particle offset ($x_0$,$y_0$). So, in effect, one ’looks up’ where the measured contrast values are found on the maps, thereby determining the actual position. Note that this method does not require sophisticated minimization algorithms or well-chosen initial parameters as the contrast function is a monotonic function for both lateral directions.

### Effect of finite bunch size

Typical particle bunches consist of large numbers $N \ggg 1$ of particles and thus have a non-negligible spatial extension in the radial and axial directions. In analogy to the expected single-ion signal, the expected voltage signal *U*(*t*) of a bunch is a superposition of all *N* individual contributions and is hence given by

6 $$\begin{aligned} U(t) =\sum _{i=1}^{N}\Gamma \left( (x_{i}(t),y_{i}(t),z_{i}(t) \right) \cdot q_{i} \cdot S, \end{aligned}$$

which can differ significantly from the expected single-particle signal (2). This refers not only to the maximum signal height, but also to its temporal structure. Previously, we have discussed the properties of such signals and how it is possible to derive quantitative information about the bunch kinetic energy, bunch length and the absolute number of particles in a bunch^21^. Here, we want to focus on the expected effect of a finite radial bunch extension on the lateral position determination.

To quantify this, we assume a bunch of 10,000 Ar$^{7+}$ ions with a kinetic energy of 2000 eV/q. Figure 3b shows the resulting contrast value $\Omega _y$ of such an ion bunch as a function of the horizontal center-of-charge distance from the central axis for different radial bunch extensions. These are characterized by Gaussian distributions of width $\sigma$ of zero, 1 mm and 2 mm, respectively.

Obviously, close to the central axis ($y_0 \approx 0$), the finite width of the ion distribution has no measurable effect as the three lines coincide. This is due to the fact that the contrast $\Omega _y$ is nearly linear in the coordinate $y_0$ close to the central axis such that a charge distribution produces nearly the same signal as a corresponding point charge at the center-of-charge position.

Observable differences occur only for larger offset distances from the axis, on the very left and right hand sides of the plot, close to the detector edges. For large $y_0$, the contrast $\Omega _y$ is no longer linear due to the finiteness of the electrode such that a charge distribution behaves differently from a point charge. For distances below $\mid y_0 \mid =2$ mm (i.e. one third of the lateral detector size) the lateral position error due to finite bunch extension is still below 50$\upmu$m and hence represents an effect on the percent level of accuracy. Due to the symmetry of the detector, the same statement is true for the horizontal direction.

The axial extent of the ion bunch has a direct influence on the temporal shape of the detected signal as depicted in Fig. 3c). The longer the ion bunch is, the higher is also the width of the signal while the peak intensity is decreasing. Nevertheless, the area below the signal is independent of the axial extent and is hence a robust measure for the ion number and to calculate the contrast ratio.

## Setup

**Figure 4:**
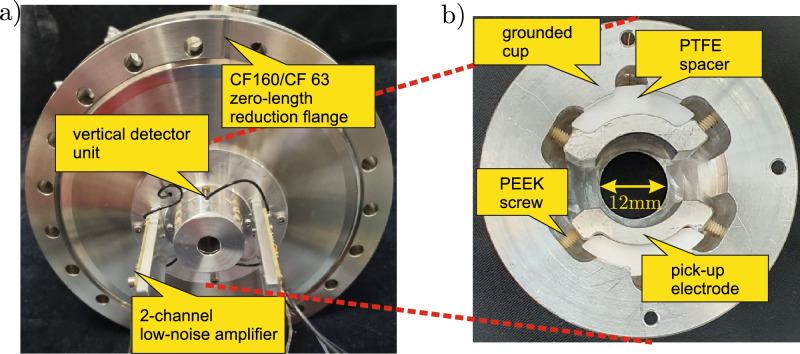
(**a**) Photo of the detector connected to a CF160/63 zero-length reduction flange. The other detector half is on the other side of the mounting ring. The two dual-channel low-noise amplifiers are connected to the vertical and the horizontal electrode pair, respectively. (**b**) Photo of one electrode pair. The two opposite electrode pairs have a distance of 15mm and are held in position by a PTFE spacer and four PEEK screws each.

### Mechanical detector design

The mechanical structure of the position-sensitive charge counter consists of two pairs of electrodes - one for the vertical and one for the horizontal position measurement. Each individual electrode is a segment of a hollow cylinder with an inner diameter of 15 mm and a length of 7.5 mm. Each segment covers an angle of 120$^\circ$ and the minimum distance between segments is 3 mm in order to reduce electronic cross-talk. Additionally, each electrode pair is covered by a grounded cup to avoid direct impact of charged particles. The effective aperture of the detector is 12 mm, determined by the opening of the grounded cup. Along the central axis, the two pairs of electrodes are separated by 45 mm. The individual electrodes are fixed with four plastics screws and are held in place by a polytetra-fluorethylene (PTFE) spacer. The overall mechanical arrangement fits into a CF63 UHV tube. Figure 4 shows two photographs of the assembly: the left one shows the detector connected to a CF160/63 zero-length reduction flange. The vertical detector part points to the front, being housed by a grounded cup with a central 12 mm hole. The horizontal part is on the other side of the mounting plate and thus not visible here. The two dual-channel low-noise amplifiers are connected to the vertical and the horizontal electrode pair, respectively. The right photograph shows a view of the vertical detection electrode pair inside the grounded cup. Two such assemblies are mounted on a mounting plate to complete the detection system.

### Electronic signal processing

The signal induced in each of the four electrodes is amplified by a two-stage room-temperature amplifier (Stahl Electronics model PR-E2) and is recorded by one channel of a four-channel oscilloscope, see Fig. 1. The amplifiers are commercially available and are optimised for frequencies between 1.5kHz and 4MHz and a capacitive load of less than 125pF. As the resulting signal height in each channel depends on the capacitance $C_e$ of the corresponding electrode and the amplification $\alpha$ of its amplifier, each channel has to be calibrated individually as detailed in^21^. For the present detector, the results are given in Table 1:

**Table 1:** Calibration data of the individual channels of the position-sensitive charge-counter: capacitance $C_e$, amplification factor $\alpha$ and sensitivity $S=\alpha /C_\text {e}$.

electrode	$C_\text {e}$ in pF	amplification $\alpha$	*S* in nV/e
left	26.84 (16)	242.09 (49)	1445 (11)
right	28.77 (17)	248.34 (51)	1383 (10)
bottom	29.94 (16)	246.48 (47)	1319 (10)
top	23.63 (12)	252.35 (36)	1711 (11)

Here, the value $C_e$ includes also the capacitance of the amplifier input which is 16pF. The capacitance of the electrodes is mainly caused by the housing close to the electrode and could be further decreased by increasing the housing inner diameter.

For electrodes on a floating potential and in close proximity to one another, in addition to the capacitance $C_e$ discussed so far, one may need to consider the cross capacitance $C_m$ from one electrode to the opposite one, as in this case an induced charge *q* will result in a voltage signal *U* in the opposing electrode. Taking the horizontal part of the detector as an example, the measured voltages $U_{right}$ and $U_{left}$ can thus be expressed as

7 $$\begin{aligned} \begin{pmatrix}U_{right} \\ U_{left}\end{pmatrix} = \begin{pmatrix} C_e &{} C_m \\ C_m &{} C_e \\ \end{pmatrix}^{-1} \begin{pmatrix}q_{right} \\ q_{left}\end{pmatrix}. \end{aligned}$$

Here, the same values of $C_e$ and $C_m$ for both electrodes are assumed in order to estimate the effect of the cross capacitance. From Eq. (7) the contrast value $\Omega _x$ can be determined to be

8 $$\begin{aligned} \Omega _x = \frac{1+C_m/C_e}{1-C_m/C_e} \Omega _x^0 = b \Omega _x^0, \end{aligned}$$

where $\Omega _x^0$ is the contrast value for $C_m = 0$. This equation shows that the cross capacitance affects the contrast $\Omega _x$ only by a constant factor *b*. From the FEM-simulation of the electrode geometry, the present cross capacitance is $C_m={-0.18}{pF}$ which results in $b\approx {0.99}$. As it is a constant factor close to unity, *b* has no influence on relative position measurements and its influence can be accounted for if absolute measurements with such high precision are required. For symmetry reasons, the same statements are true for the vertical direction.

### Ion beamline and electrostatic deflection

**Figure 5:**
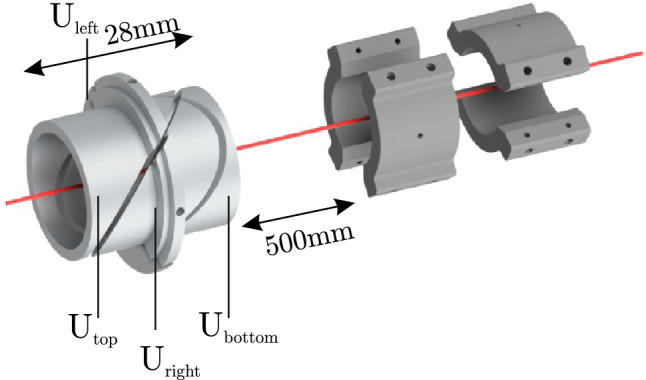
Ion optical setup for the present measurements: the ion bunches pass through a so-called Sikler lens (left) and then pervade the two electrode pairs of the detector. The devices are to scale, their separation of 500 mm is understated in the figure.

The present detector is part of the low-energy UHV beamline of the HILITE experiment^6^. The beamline connects a commercial EBIT ion source to a cryogenic Penning trap for dynamic ion capture and storage. Details of the experiment have been given in^6^. The relevant part for the present discussion is shown in Fig. 5. It consists of a so-called Sikler lens^25^ for focusing and steering of the ion beam, followed by the position-sensitive detector 500 mm downstream. In this picture, the ions from the EBIT ion source enter from the left hand side and leave for the trap towards the right hand side.

The Sikler lens is a four-fold-segmented electrostatic lens and can be used both for focusing and for small-angle steering of an ion beam by appropriate choice of the voltages applied to its four segments^25^. Together with its grounded surrounding, it has the same focusing properties as an Einzel lens when all segment voltages are identical. When different voltages are applied to the different segments, it acts as an electrostatic deflector for both lateral directions. In particular, the value of $V_{{right}}-V_{{left}}$ determines the horizontal deflection while the value of $V_{{bottom}}-V_{{top}}$ determines the vertical deflection. This is used in the following measurements to set the horizontal and vertical beam offsets from the central axis. Note that the horizontal and vertical electrode pairs of the Sikler lens have slightly different sizes, such that the horizontal deflection is different from the vertical one for the same applied voltage difference.

## Measurements

We have tested the present detector and our evaluation procedure with bunches of Ar$^{7+}$ ions at an ion kinetic energy of 1895 eV/q.

**Figure 6:**
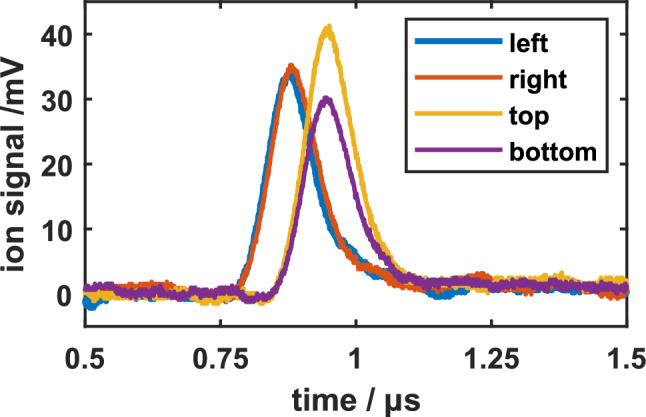
Simultaneously measured signals of all four detector channels for a single bunch of roughly 15,000 ions of Ar$^{7+}$ that has small arbitrary offsets from the central axis. The observed temporal shift between the two signal pairs of roughly 66 ns agrees well with the chosen ion kinetic energy of 1895 eV.

As an example, Fig. 6 shows the measured signals of all four detector segments resulting from a single pass of one ion bunch at some small and arbitrary lateral offset. When compared to the theoretically expected single-ion signal shown in Fig. 2, it qualitatively shows the same signal shape, and a signal area that is higher by a factor that corresponds to the number of ions in the bunch, currently about 15,000. This is in agreement with the typical ion number determined by Faraday cup measurements for identical source settings.

For subsequent position measurements, we have adjusted the initial ion beam position such that the signals of opposing detector channels where equal and thereby centered the ion beam initially. The voltage difference of opposite Sikler-lens electrodes was then scanned in order to steer the ion beam across the central ($x_0=y_0=0$) position in both lateral directions.

We have scanned the voltage differences of the vertical and the horizontal Sikler-lens electrode pairs from -60 V to +60 V, respectively. We have recorded all four signals for each ion bunch passing the detector as shown in Fig. 6. The observed temporal shift between the two signal pairs of about 66 ns agrees well with the chosen ion kinetic energy of 1895 eV/q.

**Figure 7:**
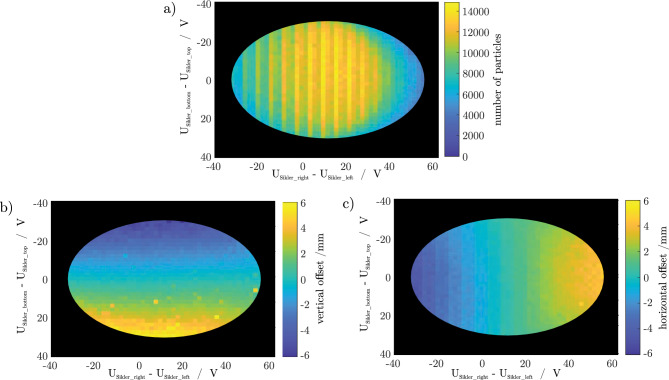
(**a**) Map of the ion number density of the ion bunch calculated from the acquired ion signals and the calibration data in Table 1. (**b**) Map of the vertical beam offset as a function of the steering voltages. (**c**) Map of the horizontal beam offset as a function of the steering voltages.

Figure 7a shows a false-color map of the observed ion number as a function of the horizontal and vertical steering voltages $V_{{right}}-V_{{left}}$ and $V_{{bottom}}-V_{{top}}$. The conversion of the observed detector signal to an ion number is done using Eq. (6) for the given ion species. As a measure we use the area below the signal. As the ion number detected by each electrode of a particular electrode pair is identical, any deviation of the area is caused by an offset from the centre in the respective direction. The position then is deduced as described in section “Lateral position determination”. Comparing now the area of the measured signal with the calculated signal of a single (see Eq. 6) ion with the determined x-y-offset gives the number of ions in this ion bunch. The lateral ion distribution has a negligible effect on the signal, as explained in section “Effect of finite bunch size” and therefore we have assumed zero beam width. Obviously, the observed ion number is highest around the center and drops off when the lateral offset is about 2mm because the ion bunches begin to get scraped by the 12 mm diameter circular opening in front of the detector. The vertical lines in the ion-number graph are caused by a periodic variation of the number of produced ions in the EBIT ion source due to fluctuation in the background pressure. In the black regions the algorithm still produces data concerning ion number and position but due to low signal-noise ratio the data here is not considered trustworthy. The resolved regions are ovals instead of circles due to the aforementioned fact that the Sikler lens deflection per voltage is slightly different for the horizontal and vertical directions.

Figure 7b and c show false-color maps of the measured ion beam displacement from the central axis as a function of the horizontal and vertical steering voltages $V_{{right}}-V_{{left}}$ and $V_{{bottom}}-V_{{top}}$. The measured values of both horizontal and vertical displacements are in the range between $-$6 mm and +6 mm, which agrees with the 12 mm diameter circular opening in front of the detector. Obviously, as desired, the horizontal steering voltage has no effect on the vertical beam position (Fig. 7b) and vice versa (Fig. 7c). In contrast, the vertical position depends linearly on the vertical steering voltage (Fig. 7b) and likewise the horizontal position depends linearly on the horizontal steering voltage (Fig. 7c).

## Summary and conclusion

We have demonstrated a position-sensitive detector for charged-particle bunches in a beamline that allows for a non-destructive measurement of particle bunch properties on a shot-to-shot basis, particularly of the lateral offset of the particles from the central detector axis. The detector design is capable of having a sensitivity of 50$\upmu$m accuracy in the detector centre and is limited by the unknown particle bunch lateral extent. The expected difference of the corresponding signal pairs for such offset is about 600$\upmu$V, which is lower than the specified noise ratio of the amplifier and can be measured with an oscilloscope with 11 bit voltage resolution. At the moment our accuracy is limited by the actual noise in our setup whose origin we assume are electromagnetic pulses by the ion source. In the present setup, the minimum ion number required for a position measurement of the stated accuracy is about 2000 Ar$^{7+}$ ions, given by the signal-to-noise ratio of the detection electronics. The absolute position accuracy in our setup is limited to 0.5mm and is given by the machining tolerances. The enhancement can be subject of further development especially by the elaboration of an adjustment procedure. By use of a third pair of electrodes where the pairs are rotated by $120^\circ$ with respect to each other, in addition to lateral particle position, it would become possible to estimate also the angle of the particle bunch trajectory with respect to the central axis of the device. Hence, one would be able to adjust the ion beam in parallel to the device axis. This allows it to be used in the framework of the HILITE experiment^6^ without restrictions, and we believe the same to be true for many applications with ion bunches from commercial and non-commercial ion sources such as EBITs. Improvements towards smaller required ion numbers can be made by noise reduction in the experimental setup, or by placing the electronics in a cryogenic region. Furthermore, the electrode geometry can be optimised for minimum capacitance, which results in a larger voltage signal for the same input charge.

The detector is UHV compatible, built in a compact fashion and can for example be installed in a standard CF63 beamline or larger, thereby allowing for a broad range of applications. The independence of the signal amplitude from the particle velocity furthermore makes it suitable for measurements across a wide range of particle kinetic energies.

## Data Availability

The data that support the findings of this study are available from the corresponding author upon reasonable request.
